# Self-care practices of Chinese individuals with diabetes

**DOI:** 10.3892/etm.2013.945

**Published:** 2013-02-05

**Authors:** YUJUAN ZHOU, LI LIAO, MEI SUN, GUOPING HE

**Affiliations:** 1 Department of Clinic Nursing, College of Nursing, University of South China, Hengyang, Hunan 421001;; 2 Department of Community Nursing, College of Nursing, Central South University, Changsha, Hunan 410083, P.R. China

**Keywords:** diabetes, self-care, diet, self-monitoring of blood glucose

## Abstract

The aim of this study was to investigate the self-care practices of Chinese individuals with diabetes. Data were collected from 163 Chinese individuals with diabetes using a one-to-one interview approach. The Chinese version of the Summary of Diabetes Self-Care Activities (SDSCA) was used to assess diabetes-related knowledge and self-care practices. The majority of participants were aware of the importance of self-care in managing diabetes. However, only 70 participants (43%) scored >50% in the diabetes-related questionnaires. Mean fasting blood glucose (FBG) levels were higher (P<0.04) for participants who had extra meals per day (46%). The majority of participants took oral hypoglycemic agents (OHAs; 60.1%) and some were also treated with OHA-insulin combination therapy (17.8%). Participants with medication adherence (52%) tended to have lower FBG levels. Only 13% of participants practiced self-monitoring of blood glucose (SMBG). The predictors of a knowledge deficit or poor self-care were a low level of education (P<0.01) or old age (older than 53 years old; P=0.002). Deficits in diabetes-related knowledge and self-care practices existed among the majority of patients with suboptimal blood glucose control. The understanding of the importance of self-care practices requires improvement in individuals with diabetes. The development of effective education strategies to improve the awareness of self-care practices by Chinese individuals with diabetes is necessary.

## Introduction

Diabetes mellitus (DM) is a chronic disease with severe complications and high mortality. Diabetes is a leading cause of kidney failure, new blindness in adults, and leg and foot amputations unrelated to injury. It is also a major cause of heart disease and stroke, which is associated with persistent hyperglycemia, hyperlipidemia and glycosylation. The number of cases of DM is escalating, making it a global public health concern, particularly in Asia (1), which may be due to rapid changes in nutritional habits, aging and urbanization. It is estimated that ∼42.3 million Chinese individuals will have diabetes by 2030 (2). Numerous studies indicate that the risk of diabetes and its complications may be reduced through appropriate management and treatments (3). However, it has been reported that one of the causes of diabetes complications is a lack of self-care by patients with diabetes (4).

The importance of self-management for young or old individuals with diabetes has been noted by a number of researchers (5–7). However, the self-management of individuals is often influenced by numerous biological and psychosocial factors. It is a challenge for individuals with diabetes to self-manage their condition to prevent or delay the onset of complications, for example, by making lifestyle changes to diet and physical activity levels (8). The American Association of Diabetes Educators (AADE) focus on seven key behaviors, including healthy eating, being active, monitoring, taking medication, problem solving, healthy coping and reducing risks, which are called the AADE7™ self-care behaviors and may promote successful self-management. Thus, self-care tasks have been defined as a set of management strategies undertaken to manage personal illness (9). Among these management strategies, dietary intake, medication use, physical activity and self-monitoring of blood glucose (SMBG) are the four main cornerstones of overall diabetes management.

The successful management of diabetes relies on the individual performing self-care activities designed to control symptoms and avoid complications. McCollum *et al* (5) emphasized that effective self-care is an essential component of diabetes care. Diabetes-related confidence in the ability to perform the necessary self-care regimen is a crucial outcome measurement for educational programs. Ninety-five percent of diabetes treatment relies on self-care behaviors (10). Regardless of the diabetes type, diabetic patients must adjust their behavior and follow prescribed treatments to prevent diabetic complications, which may be potentially fatal, particularly for older individuals (11).

The Summary of Diabetes Self-Care Activities (SDSCA) has been used extensively with various ethnic populations to measure diabetes self-management. The revised version of the SDSCA measure (12) is a widely used tool for evaluating the frequency of diabetes self-care tasks performed by the participants. Despite the abundance of studies on self-care practices, there is limited information available on the self-care practices of individuals with diabetes in China. In this study, we used the Chinese version of SDSCA, since it has demonstrated acceptable reliability (0.62–0.87) and validity (13), to investigate the self-care practices of individuals with diabetes living in central China.

## Materials and methods

### Participants

A descriptive study was used to investigate the self-care practices of 163 patients recruited between January and May 2010, from the First Hospital of University of South China (Hunan, China). The participants were non-pregnant adults including both genders and diagnosed as either Type 1 or Type 2 with a medical record indicating suboptimal glycemic control over the last year. It was also required that patients had neither hearing or visual impairment nor physical or mental impairment, which may interfere with answering the questionnaire independently. It was previously observed that fasting blood glucose (FBG) is associated with increased micro- and macro-vascular complications (14). Accordingly, suboptimal glycemic control was defined as a mean of at least three FBG levels of >7 mmol/l in the previous year of laboratory samples, due to the limited availability of participant HbA1c results.

### Sampling population

The study included 163 participants (78 females and 85 males) with an average age of 53.1 years. Among them, 4 had Type 1 diabetes and 159 had Type 2 diabetes. The average duration of diabetes was 9.7 years and the mean FBG was 11.8 mmol/l.

### SDSCA measurement

The Chinese version of the SDSCA (13) was used to measure various diabetes self-care activities. Scores were calculated for each area creating five subscales: i) general diet, ii) eating a diet high in fruits/vegetables and low in high-fat foods, iii) daily activity, non-leisure and leisure activity, using three categories namely ‘most active’, ‘moderately active’ and ‘least active’, iv) taking recommended medications, v) glucose self-monitoring. Using a continuous scale ranging from 0–7, the numerical scoring of items was based on the number of days of the week that the behavior was performed; the item scores were averaged resulting in an overall score for each self-care activity. Particularly, physical activities were evaluated by the intensity of total daily activity, including non-leisure and leisure activities, using three categories namely ‘most active’, ‘moderately active’ and ‘least active’.

### Ethical considerations

Ethical approval was received from the Joint Ethics Committee of the Central South University Health Authority (Changsha, China). The researchers provided participants with an information sheet and explained the details of the study prior to obtaining written informed consent. The participants were guaranteed of anonymity, confidentiality and penalty-free withdrawal allowance.

### Procedure

After gaining informed consent, the investigator read the questionnaire to the subjects and recorded the responses. The interviewer bias was managed by discussion between the two investigators. Actual sizes of spoons, plates, bowls and photos of fruits and food servings were used to enhance dietary recall and improve reliability. To ensure consistency, the investigator converted the reported food intake to appropriate serving sizes and recorded the frequency of consumption after checking this with the subjects. Medicine adherence measurement was performed by a visual analog scale. Oral diabetic medications and insulin were used to enhance physical activity and medication recall, respectively.

### Statistical analysis

The Statistical Package for the Social Sciences (SPSS, Inc., Chicago, IL, USA) 13.0 for Windows was used for statistical analysis. Demographic data were analyzed using the frequencies method of descriptive statistics. Reliability was assessed using Cronbach’s α coefficient and the coefficient in this study was 0.74. The differences and associations between variables were analysed by independent sample Student’s t-tests, Chi-square test, one-way ANOVA, Mann-Whitney U test, Kruskal-Wallis test and Pearson’s or Spearman’s product-moment correlation. Computations evaluated the magnitude of association between baseline and test-retest and between SDSCA scales and criterion validity coefficients. P<0.05 was considered to indicate a statistically significant result.

## Results

### Demographic data

All 163 participants completed the questionnaires. As listed in Table I, the mean age of these participants was 53.1 years. The average duration of diabetes was 9.7 years (range, 3.3–16.1 years). Eighty-eight participants had spent ≤6 years in education. Diabetes knowledge means that patients understand the pathophysiology of diabetes and the principles of diabetes management. The mean score for total knowledge was 7.4±2.7, with 43% of the participants achieving >50%. As for dietary knowledge, however, a mean score of 2.4±1.2 was achieved, with only 8% of participants scoring >50% of the maximum. Notably, participants younger than 53 years old (F=6.8, P=0.002) with more education (χ^2^=8.3, P=0.012) achieved statistically significantly higher scores for diabetes knowledge.

### Diet self-care

Sixty-six percent of participants (n=75) consumed four or more meals a day. In addition to the three main meals, 48% consumed more than two carbohydrate portions or 30 g of carbohydrate per snack. Only 12% of participants consumed sweetened food in their daily meal plans. The mean of FBG for all participants was 11.8±2.8 mmol/l, The mean FBG of participants eating four or more meals (12.7±1.90 mmol/l) was significantly higher than that of those eating fewer than four meals (11.1±1.70 mmol/l; P=0.01). In addition, 52% of participants had consumed sweetened food or drinks more than three times during the preceding week. The ∼74% of the participants who did not include the sweetened intake in their daily meal plans showed a significantly higher mean FBG (13.1±1.42 mmol/l) than those who consumed sweetened food in their daily meal plans (11.4±1.20 mmol/l; P=0. 03). In addition, 26% of the participants consumed fruits daily and 7% of the participants had not eaten any during the preceding week.

### Exercise self-care

Physical activities are behavioral interventions that individuals undertake to manage the disease. The activity levels of the participants are shown in Table II. (The items of this table were set according to a previous study with certain modifications (15). Overall, 56% of participants were least active in total daily life (leisure and non-leisure activities). Furthermore, while 44% of participants were least active in non-leisure activities, only 4% of participants were most active. Similarly, 60% of the participants were least active in leisure activities and the remainder of participants (40%) reported taking regular exercise (34%) or being most active (6%) during their leisure hours.

The participants aged ≥53 years were less active than the younger participants (χ^2^=4.61, P=0.032) in leisure activities, rather than non-leisure activities. Therefore, the level of exercise undertaken was relevant to the individual’s physical capability. Participants with a mean FBG level <9 mmol/l were more active than those with a mean FBG >11.0 mmol/l in both leisure (F=3.71, P=0.02) and total daily activities (F=3.19; P=0.02). The male participants appeared to be more active than the female participants in both leisure and non-leisure physical activities, but this was not statistically significant (P>0.05).

### Medication self-care adherence

The disease duration for all participants was 0.3–16 years (mean ± SD, 9.7±6.4 years). Ninety-three participants (57.1%) had diabetes for >9 years. As shown in Table III, the majority of participants were treated with oral hypoglycemic agents (OHAs; 60.1%) and OHA-insulin combination therapy (17.8%). The OHA medication adherence rate was 81±14.3%, with 47% of subjects achieving the level defined as adherence (≥90% of their daily prescribed medication). Compared with the other participants who were not in hospital, the demographic predictors of higher medication adherence were participants from the hospital, managed solely on insulin, versus participants on OHAs or combination therapy (86% versus 47% and 41%, χ^2^=18.4, P<0.001). Those participants with secondary education and above were also more likely to adhere to their prescriptions (χ^2^=12.2, P=0.01).

### Self-monitoring of blood glucose

The participants were generally aware of the importance of self-care. Specifically, 91%, 97%, 86% and 58% of participants realized the significance of diet, medication, exercise and SMBG, respectively. However, only 21 participants (13%) practiced SMBG and 5 (3%) had tested four or more times during the preceding week. In addition, 7 of the 21 participants had modified their diet based on blood glucose readings, medication or exercise.

### Previous education received

The percentages of participants who had received advice on diet, exercise and SMBG were 92%, 84% and 53%, respectively. The majority of participants from the hospital settings reported that they had been advised on diet and SMBG. However, 61% of the participants did not put the advice into effect and none recalled the advice concerning carbohydrate intake. Furthermore, only 43% of the participants practiced SMBG. The percentage of participants who had received advice on exercise according to the recommended guidelines was only 32%.

## Discussion

Diabetes self-care is an essential component of diabetes care. In this study, we investigated the current situation of self-care practices of diabetes in China to enhance the understanding of the factors that contribute to efficient self-care practices of diabetes. It has been reported that an essential basis for effective diabetes management is diabetes-related knowledge (16), including knowledge with regard to the correct diet, medication and SMBG. However, due to the lack of trained diabetes educators in China, patients with diabetes receive only limited diabetes-related information from physicians, books, TV programs or other media. In this study, only 43% of the participants scored >50% in the questionnaires, indicating that these participants with sub-optimal glycemic control pay insufficient attention to diabetes and self-care practices.

We observed that a low level of education and old age of patients with diabetes may be predictors for a knowledge deficit and poor self-care, which is in accordance with previous studies (17–19). Due to the low functional health literacy of individuals older than 53 years, these individuals are likely have less comprehension of the previous knowledge received on diet and exercise. Notably, patients who were on insulin treatment generally have higher levels of knowledge. This may be due to the complexity of insulin treatment, which may cause participants to seek information more actively. By the time an individual starts insulin therapy, diabetes-related complications are already likely to be present due to poor blood sugar control.

The association between carbohydrate consumption and sub-optimal glycemic control was also evaluated in this study, using meal frequency, dietary knowledge and content of food intake. Studies have shown that it is acceptable for a healthy diet if sugar or sucrose constitutes no more than 10% of the total caloric intake or 50 g per day (20,21). However, sugar or sucrose should be consumed as a substitute for other carbohydrate in the total daily calories (22). In the current study, 64 out of the 163 participants had regular snacks besides three main meals and 48% consumed more than two carbohydrate portions per snack. A weak positive correlation was observed between the number of meals consumed and the mean FBG (P=0.01). In addition, 52% of participants had a statistically significantly higher mean FBG due to the intake of more than three sweetened foods or drinks during the preceding week, which was consistent with the findings of previous studies (23).

It has been revealed that glucose control is sensitive to variations in the adherence to medication and SMBG (24). We observed that the medication adherence rate was lower than previously reported (25,26). This may be due to our definition of ≥90% adherence, instead of 80% in previous studies. Of the participants, 60.1% were treated with OHAs and 17.8% combined OHAs with insulin injections, and the OHA medication adherence rate was 81±14.3%. However, participants who only used insulin achieved greater medication adherence than participants on OHAs or combination therapy (P<0.001). The level of education appeared to play a significant role in medication adherence (P=0.01), in accordance with a previous study (27). As for SMBG, a consistent barrier is finance, as has been previously reported (28). Few patients in China were able to afford to use blood glucose self-monitoring equipment, and so had their blood glucose levels checked at the hospital. Therefore, only 13% of participants practiced SMBG, and only 3% participants had tested four or more times during the preceding week.

Physical activity is a behavioral intervention that a patient undertakes to care for the disease (Table II). However, in the current study, the majority of the participants (56%) did not take sufficient exercise and only 6% had sufficient physical activity for glycemic control. Notably, females were less active than males, and 57% of the participants aged ≥53 years were less active than the younger participants. We observed that the less active participants had a higher mean FBG, which is consistent with the findings of previous studies (29).

Although the present study provides important insights concerning the current situation of self-care practices of adults with diabetes in China, several limitations should be noted. The main limitation is that this study was conducted using self-reporting, rather than the direct observation of self-care practices. The use of non-familiar instruments and specific entry criteria may also present a threat to internal validity, and potential study bias may be created by the judgment of two different reviewers. Furthermore, the study was set within one province in China, thus replication of this study with appropriate probability sampling is required to improve its general applicability. It would be worthwhile in the future to investigate the discrepancy between the perceived importance of self-care practices and actual behavior by using a triangulated research methodology, including a qualitative approach, which would substantially enhance our understanding of the self-care practices of patients with diabetes.

Despite the awareness of the importance of self-care in managing diabetes, the majority of the patients had difficulty understanding and practicing self-care. This is likely due to limited education, old age and a deficit in diabetes-related knowledge. However, it should be noted that the fundamental reason for the current situation may be the lack of facilities and certified educators of diabetes in China. By enhancing the understanding of the factors that contribute to self-care practices, this study has laid the foundation for future studies to improve the situation of diabetes education and management in China.

By using the Chinese version of SDSCA, we observed that deficits in diabetes-related knowledge and self-care practices exist among the majority of the patients with suboptimal blood glucose control. The understanding of the importance of self-care practices by individuals with diabetes requires improvement. The development of effective education strategies to improve the consciousness of self-care practices in all Chinese individuals with diabetes is necessary.

## Figures and Tables

**Table I:** Demographic data of participants (n=163).

Variable	Mean (%)
Age (years ± SD)	53.1±14.64
Duration of diabetes (years ± SD)	9.7±6.4
Mean fasting blood glucose (mmol/l ± SD)	11.8±2.8
Gender	
Female	78 (48)
Male	85 (52)
Ethnicity	
Chinese	123 (76)
Other	40 (24)
Type of diabetes	
Type 1	4 (2)
Type 2	159 (98)
Education	
Illiterate	15 (9)
Elementary school	73 (45)
Junior high school	30 (18)
Senior high school	27 (17)
College and above	18 (11)
Living status	
Living with family	147 (90)
Living without family	16 (10)
Type of treatment	
Diet and exercise	0 (0)
Oral hypoglycemic agents	98 (60)
Insulin	36 (22)
Oral + insulin (combination therapy)	29 (18)

**Table II:** Physical activity levels of participants.

Type of activity	Category of activity	Definition of category	Activity score	No. of participants	% of participants
Non-leisure	Least active	Almost all the time sitting, less than half of the time standing or walking, seldom carrying heavy objects and travels by car or motorbike.	6–13	71	44
	Moderately active	Sitting, standing and walking about half of the time, sometimes carrying heavy objects, uses public transport during non-leisure hours.	14–21	84	52
	Most active	Almost no time sitting, almost all the time standing or walking, most of the time carrying heavy objects, uses public transport, cycles or walks between home and other activities.	22–29	8	4
Leisure	Least active	Never or seldom walking around the house, sometimes sitting down, no gardening or regular exercise program	3–9	97	60
	Moderately active	Sometimes gardening, walking around the house, sitting down to watch TV, inconsistent exercise program with minimum intensity.	10–20	56	34
	Most active	Most of the time walking around the house, gardening, seldom sitting down, exercises regularly with moderate intensity on ≥5 days a week for >30 min each day.	21–31	10	6
Total	Least active	Least active in both non-leisure and leisure activities as defined.	9–23	91	56
	Moderately active	Moderately active in both non-leisure and leisure activities as defined.	24–42	65	40
	Most active	Most active in both non-leisure and leisure activities as defined.	43–60	7	4

**Table III:** Medication adherence rate according to type of treatment.

Type of treatment	N	Medication adherence (%) mean (SD)	Participants with >90% medication adherence rate (%)
Oral hypoglycemic agents	87	81 (14.3)	47
Insulin	36	92 (13.0)	86
Oral + insulin	29	83 (14.2)	41
Overall type of treatment	163	85 (15.2)	52
